# Cisplatin and carboplatin result in similar gonadotoxicity in immature human testis with implications for fertility preservation in childhood cancer

**DOI:** 10.1186/s12916-020-01844-y

**Published:** 2020-12-04

**Authors:** Melissa D. Tharmalingam, Gabriele Matilionyte, William H. B. Wallace, Jan-Bernd Stukenborg, Kirsi Jahnukainen, Elizabeth Oliver, Anne Goriely, Sheila Lane, Jingtao Guo, Bradley Cairns, Anne Jorgensen, Caroline M. Allen, Federica Lopes, Richard A. Anderson, Norah Spears, Rod T. Mitchell

**Affiliations:** 1 MRC Centre for Reproductive Health, The Queen’s Medical Research Institute, The University of Edinburgh, 47 Little France Crescent, Edinburgh, EH16 4TJ Scotland, UK; 2 KK Women’s and Children’s Hospital, Bukit Timah Rd, 100, Singapore, 229899 Singapore; 3 Edinburgh Royal Hospital for Sick Children, 9 Sciennes Road, Edinburgh, EH9 1LF Scotland, UK; 4 NORDFERTIL Research Lab Stockholm, Childhood Cancer Research Unit, Department of Women’s and Children’s Health, Karolinska Institutet and Karolinska University Hospital, Stockholm, Sweden; 5 Division of Haematology-Oncology and Stem Cell Transplantation, Children’s Hospital, University of Helsinki, Helsinki University Central Hospital, Helsinki, Finland; 6 Radcliffe Department of Medicine, MRC Weatherall Institute of Molecular Medicine, University of Oxford, Oxford, OX39DS UK; 7 Department of Paediatrics and Child Health, Oxford University Hospitals NHS Foundation Trust, and Nuffield Department of Womens and Reproductive Health, University of Oxford, Oxford, UK; 8 Section of Andrology, Division of Urology, Department of Surgery, University of Utah School of Medicine, Salt Lake City, UT USA; 9 Howard Hughes Medical Institute, Department of Oncological Sciences and Huntsman Cancer Institute, University of Utah School of Medicine, Salt Lake City, UT USA; 10 Department of Growth and Reproduction, Copenhagen University Hospital (Rigshospitalet), Blegdamsvej 9, 2100 Copenhagen, Denmark; 11 Biomedical Sciences, University of Edinburgh, Edinburgh, EH8 9XD UK

**Keywords:** Human, Testis, Cisplatin, Germ cell, Fertility, Prepubertal, Foetal, Xenograft

## Abstract

**Background:**

Clinical studies indicate chemotherapy agents used in childhood cancer treatment regimens may impact future fertility. However, effects of individual agents on prepubertal human testis, necessary to identify later risk, have not been determined. The study aimed to investigate the impact of cisplatin, commonly used in childhood cancer, on immature (foetal and prepubertal) human testicular tissues. Comparison was made with carboplatin, which is used as an alternative to cisplatin in order to reduce toxicity in healthy tissues.

**Methods:**

We developed an organotypic culture system combined with xenografting to determine the effect of clinically-relevant exposure to platinum-based chemotherapeutics on human testis. Human foetal and prepubertal testicular tissues were cultured and exposed to cisplatin, carboplatin or vehicle for 24 h, followed by 24–240 h in culture or long-term xenografting. Survival, proliferation and apoptosis of prepubertal germ stem cell populations (gonocytes and spermatogonia), critical for sperm production in adulthood, were quantified.

**Results:**

Cisplatin exposure resulted in a significant reduction in the total number of germ cells (− 44%, *p* < 0.0001) in human foetal testis, which involved an initial loss of gonocytes followed by a significant reduction in spermatogonia. This coincided with a reduction (− 70%, *p* < 0.05) in germ cell proliferation. Cisplatin exposure resulted in similar effects on total germ cell number (including spermatogonial stem cells) in prepubertal human testicular tissues, demonstrating direct relevance to childhood cancer patients. Xenografting of cisplatin-exposed human foetal testicular tissue demonstrated that germ cell loss (− 42%, *p* < 0.01) persisted at 12 weeks. Comparison between exposures to human-relevant concentrations of cisplatin and carboplatin revealed a very similar degree of germ cell loss at 240 h post-exposure.

**Conclusions:**

This is the first demonstration of direct effects of chemotherapy exposure on germ cell populations in human foetal and prepubertal testis, demonstrating platinum-induced loss of all germ cell populations, and similar effects of cisplatin or carboplatin. Furthermore, these experimental approaches can be used to determine the effects of established and novel cancer therapies on the developing testis that will inform fertility counselling and development of strategies to preserve fertility in children with cancer.

**Supplementary information:**

The online version contains supplementary material available at 10.1186/s12916-020-01844-y.

## Background

Survival rates for children with cancer have increased dramatically over recent decades, and currently, more than 80% of those affected are expected to survive their disease into adulthood [1]. However, the long-term impacts of cancer treatment remain a major concern for this patient population. The mainstay of cancer treatment includes chemotherapy and radiotherapy, both of which have the potential to damage healthy tissues resulting in significant long-term morbidity [2]. An association between chemotherapy exposure and effects on the reproductive system in male and female childhood cancer survivors is well-recognised and this may result in infertility in adulthood [3, 4]. Alkylating agents (e.g. cyclophosphamide and procarbazine) are reported to be amongst the most highly gonadotoxic chemotherapeutic compounds in both males and females, and cumulative exposure to these agents can be used to estimate the risk of gonadal damage in patients due to receive such therapies [5]. However, these models do not include non-alkylating agents that may also contribute to gonadotoxicity, nor are they able to determine the direct effects of chemotherapeutic agents on the gonad.

Major differences exist between the prepubertal and adult testis in humans, especially in terms of germ cell populations [6]. Spermatogenesis does not start until after puberty and therefore sperm are not present. The germ cell populations in the prepubertal testis are predominantly spermatogonia, expressing MAGEA4 protein. This includes a sub-population of spermatogonial stem cells (SSC), which express UTF1 [7, 8]. In human foetal and infantile testis, an additional population of undifferentiated germ cells (gonocytes), which express AP2γ, are present. Gonocytes differentiate into (pre)spermatogonia over the course of foetal and early postnatal life [6]. Therefore, future fertility is dependent on differentiation from gonocyte to spermatogonia in infancy and subsequent survival of the SSC population during childhood.

Platinum-based chemotherapeutic drugs are widely used in paediatric oncology for the treatment of solid tumours including neuroblastoma, germ cell tumours and osteosarcoma [5, 9, 10]. These agents are considered to be cell-cycle independent and act to form DNA adducts, which elicit apoptosis and cell death [11]. Cisplatin is the most frequently used platinum-based compound, whilst carboplatin may be used as an alternative to cisplatin as it is considered to have a lower risk of ototoxicity and nephrotoxicity [12], although relative gonadotoxicity remains to be determined.

No previous studies have been conducted to test the effects of platinum-based chemotherapy on human testicular tissues. However, experimental systems have recently been developed to assess the direct impact of pharmaceuticals on immature human testicular tissues including short- (in vitro) and long-term (testicular xenograft) effects of acute exposure [13, 14]. These experimental approaches utilise human foetal testis, which contain the major germ cell populations of the childhood testis (gonocytes and spermatogonia), and can therefore be used to model the acute effects of chemotherapy exposure and potential for recovery of immature human testicular tissue.

In the present study, we aimed to determine the effects of exposure to clinically-relevant concentrations of platinum-based chemotherapy on germ cells of the immature prepubertal human testis using human foetal and prepubertal tissues. We also aimed to investigate the relative gonadotoxicity of cisplatin compared to that of carboplatin.

## Methods

### Experimental design

**Fig. 1:**
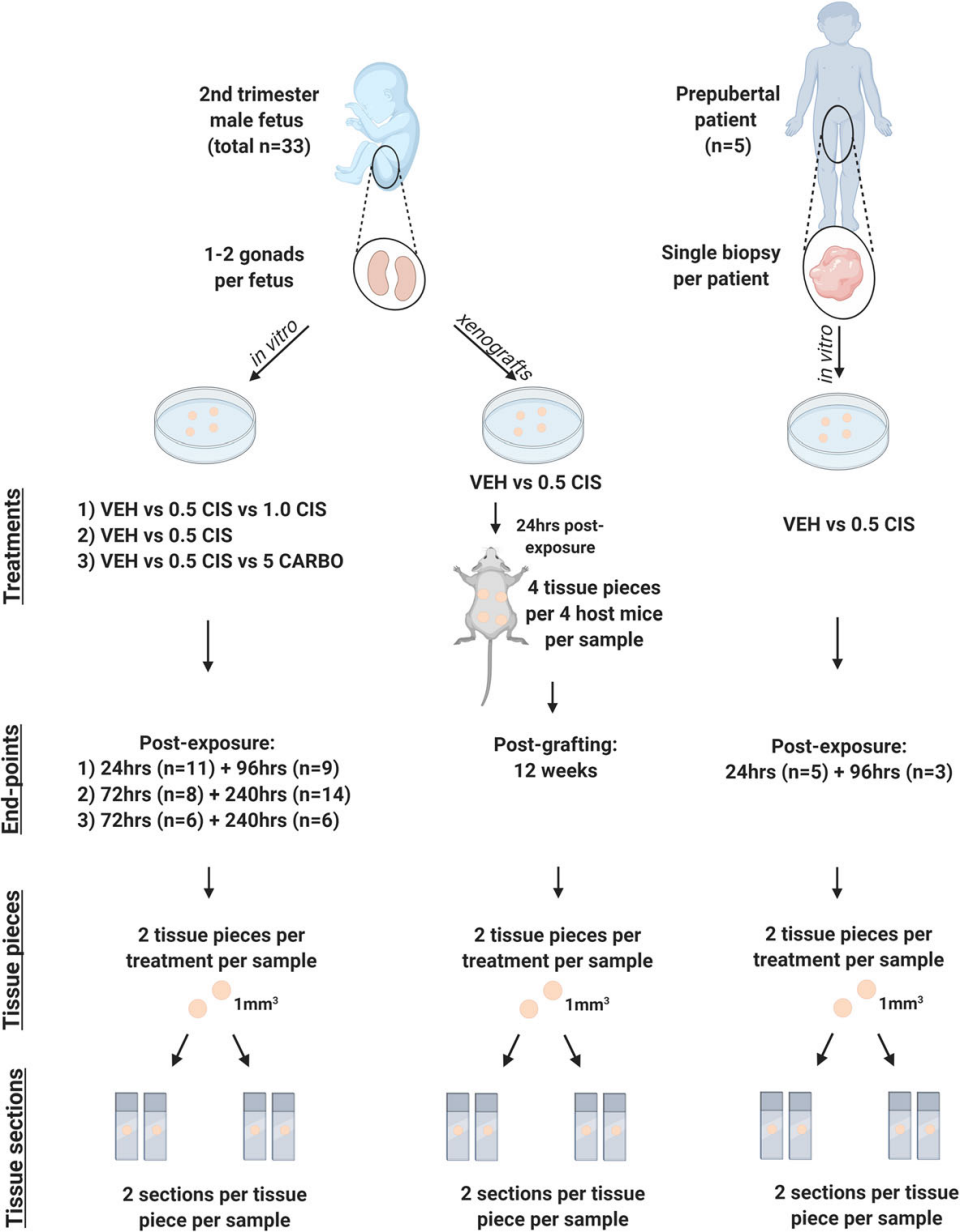
Overview of experimental setup. Designed using BioRender.com. Abbreviations: VEH, vehicle; 0.5 CIS, cisplatin (0.5 μg/ml); 1.0 CIS, cisplatin (1.0 μg/ml); CARBO, carboplatin (5 μg/ml)

Given that childhood cancer survivors are reported to have lower fertility rates in adulthood, we aimed to understand the effects of exposure to platinum-based chemotherapy agents (cisplatin and carboplatin) on key germ cell populations in immature human testis. We performed controlled laboratory experiments using both in vitro (short-term) and xenograft (long-term) models (Fig. 1). Experimental protocols were designed prior start of experiments. Immature human testicular tissue pieces were exposed to clinically relevant chemotherapy doses based on human plasma serum levels obtained from paediatric cancer patients. Sample size was determined based on previous studies using human tissue (at least *n* = 5 for each experiment).

Initial experiments using human foetal tissues investigated the effects of cisplatin-exposure on germ cells using two drug concentrations (0.5 and 1.0 μg/ml) and two time-points post-exposure (24 and 96 h). Further experiments were performed to determine the germ cell effects of cisplatin-exposure (0.5 μg/ml only) at intermediate (72 h) and long-term (240 h) time-points. Some of the in vitro cultured tissue pieces were randomly selected at 24 h post-exposure and subsequently xenografted for 12 weeks. Reciprocal grafts of control- or cisplatin-exposed tissue were grafted into the right and left dorsal flank of the same mouse, respectively. In addition, a comparison between exposure to cisplatin (0.5 μg/ml) and therapeutic equivalent concentrations of carboplatin (5 μg/ml) was investigated at 72 and 240 h post-exposure. Due to the limited amount of available tissue, the effects of cisplatin-exposure on germ cells in prepubertal human testicular tissue were investigated using a single concentration of cisplatin (0.5 μg/ml) at two time-points post-exposure (24 and 96 h).

All data were analysed once the study was completed and no outliers were excluded. To compare the effect of treatment versus vehicle controls, we analysed at least two tissue sections from two replicates for each treatment. Endpoints included the quantification of key germ cell populations and assessment of proliferation and apoptosis. Investigators performing the quantification were blinded to the treatment group.

### Study approval

Ethical approval for use of human foetal tissues for research was obtained from the South East Scotland Research Ethics Committee (LREC08/S1101/1), NRES committee North East – Newcastle and North Tyneside 1 (08/H0906/21+5) and NRES Committee London – Fulham (18/10/0822), and written informed consent was given by the women. Ethical approval for collection and use of human prepubertal testis tissues in research was obtained from the South East Scotland Research Ethics Committee (13/SS0145) and Oxford University Hospitals NHS Foundation Trust (2016/0140). Written informed consent was obtained from patients’ parents/guardians and/or the patient themselves (where appropriate).

For studies involving animals, specific approval, including ethical approval, was given by the UK Home Office. All procedures carried out were by the University of Edinburgh Bioresearch and Veterinary Services and performed in accordance with the Animal (Scientific procedures) Act 1986.

### Tissue collection

#### Human foetal testicular tissues

Testes from second-trimester human foetuses (14–22 weeks gestation; total *n* = 33) were obtained after medical and surgical termination of pregnancy as described previously [6]. None of the terminations were due to foetal abnormalities. Ultrasound scan and subsequent direct measurement of foot length were used to determine gestational age. Sex was determined by PCR for the male-specific gene *SRY*.

For studies involving animals, specific approval, including ethical approval, was given by the UK Home Office and all procedures carried out were in accordance with the Animal (Scientific procedures) Act 1986.

#### Prepubertal human testicular tissues

Prepubertal testicular tissues (*n* = 5 patients; aged 1, 1, 8, 11 and 12 years) were obtained from chemotherapy-naïve patients undergoing a testicular biopsy for fertility preservation at the Royal Hospital for Sick Children in Edinburgh and John Radcliffe Hospital in Oxford. A small portion (~ 10%) of the biopsy was donated for research purposes. Testicular tissues from Edinburgh were collected and immediately transported in Nutristem® hSEC XF Medium (Biological industries) supplemented with 1% penicillin/streptomycin (Sigma-Aldrich). Testicular biopsies from Oxford were transported overnight in Hank’s Balanced Salt solution (HBSS) with 10% human serum albumin.

### Hanging drop culture system

Human foetal and prepubertal tissue was utilised for in vitro experiments in a hanging drop culture system [15]. Testicular tissue was cut into small pieces (~ 1 mm^3^) and placed into 30 μl droplets of appropriate media (for foetal tissue: Alpha MEM (Lonza); 10% foetal bovine serum, 1% penicillin/streptomycin, 1% non-essential amino acids, 2 mM L-glutamine, 2 mM sodium pyruvate and 1% insulin transferrin selenium (ITS) (all Sigma-Aldrich); for prepubertal tissue: Alpha MEM (Lonza) and 10% Knockout Serum Replacement (KSR; Gibco)) and cultured at 37 °C and 32 °C, respectively, under 5% CO_2._ Tissue pieces were cultured for 1–3 days (subject to the exact timing of the tissue arrival) prior to exposure to cisplatin (Sigma-Aldrich) or vehicle control (ddH_2_O) for 24 h. Tissue pieces were then transferred to fresh media without cisplatin and cultured until 24, 72, 96 and 240 h post-cisplatin exposure. In a separate set of culture experiments, tissues were exposed for 24 h to either cisplatin (0.5 μg/ml), carboplatin (5 μg/ml; Calbiochem) or vehicle control (ddH_2_O) for 24 h and the experiment was ended at 72 and 240 h post-exposure. In some cases, there was sufficient material from a biopsy sample to allocate to more than one experimental setup.

### Human tissue xenografting

Testicular tissue pieces from human foetuses (*n* = 4) that were cultured in vitro were used for xenografting at 24 h post-cisplatin or vehicle exposure. As previously described [16], tissue fragments were grafted subcutaneously into host adult CD1-nude mice using a 13-gauge cancer implant needle (Popper and Sons). For each foetal sample, a total of 4 pieces (~ 1 mm^3^) of testicular tissue was inserted under the dorsal skin of the host mouse, with random allocation of two vehicle controls and two 0.5 μg/ml cisplatin-treated pieces along either side of the midline. A total of 4 mice were grafted per foetal sample, and the mice were maintained for 12 weeks post grafting before retrieval of the tissue. Mice were housed in the same location and exposed to 12 h light/dark cycles and room temperature, ~ 20–25 °C. Mice were administered 100 μl of 20 IU human chorionic gonadotrophin (hCG) (Ovitrelle, Merck Serono) beginning a week after xenografting which was continued three times a week until the end of the experiment. One mouse was culled before the end of the experiment due to poor health and was not included in the analysis. For xenograft retrieval, host mice were culled by inhalation of CO_2_ and cervical dislocation. Weight of host mouse body and retrieved grafts were recorded.

### Tissue processing

At the end of each experiment, tissue pieces were fixed in Bouin’s fluid (Clin-Tech) for 1 h and transferred to 70% ethanol. Post fixation, samples were paraffin-embedded, 5 μm sectioned and assessed by H&E using standard protocol or used for immunohistochemistry as described below. Only samples that showed healthy tissue morphology (defined tubules, minimal apoptosis and germ cell presence) in pre-culture and vehicle controls were included in the analyses. Two sections from two replicates for each treatment were stained and analysed.

### Immunohistochemistry

Double colourimetric immunohistochemistry was performed to detect gonocytes and spermatogonia (details of primary antibody in Additional File 1: Table S1).

Sections were dewaxed by incubating in xylene (BDH Prolabo®, VWR Chemicals) twice for 5 min each. Tissue slides were rehydrated in alcohol series (100% twice, 95%, 80% and 70%) for 20 s and thoroughly rinsed in tap water. Heat-induced epitope retrieval of all slides was carried out in a pressure cooker containing 0.01 M citrate buffer. Tissue sections were rinsed in water and blocked with 3% (v/v) hydrogen peroxide in methanol for 15 min on a rocker at room temperature. To minimise cross-reactivity, tissue sections were incubated with blocking agent (consisting of 20% appropriate animal serum in Tris-buffered saline (TBS, 0.01 M Tris, 0.85% sodium chloride, pH 7.4) and 5 g of bovine serum albumin (BSA, Sigma-Aldrich, GmbH)) for 30 min at room temperature. Primary antibody diluted in blocking agent was added to tissue sections for overnight incubation at 4 °C.

To detect the primary antibody, slides were incubated with peroxidase (ImmPRESS, Vector Laboratories) for 30 min at room temperature. Each incubation step was followed by two 5-min washes in TBS. To visualise the first antibody, 3,3-diaminobenzidine (DAB, Vector Laboratories) was added and colour development (usually up to 3 min) was monitored microscopically and stopped by washing in deionised water. Prior to the second antibody detection, antigen epitopes were retrieved by placing slides in pre-heated 0.01 M citrate buffer and boiling for 2.5 min using a microwave. Once cooled, slides were blocked with appropriate normal serum/TBS/BSA for 30 min at room temperature, followed by an overnight incubation at 4 °C with subsequent primary antibody diluted in normal serum/TBS/BSA.

To detect the second antibody, tissue sections were incubated with alkaline phosphatase (ImmPRESS, Vector Laboratories) for 30 min at room temperature, visualised by adding FastBlue substrate (Vector Laboratories), and the reaction was stopped by rinsing in deionised water. Slides were mounted with PermaFluor aqueous medium (Lab Vision™, Thermo Scientific). Images were taken on Axio Scan.Z1 slide scanner (Carl Zeiss Microscopy, GmbH) at × 20 magnification.

In addition, each experiment involved positive (all reagents added) and negative controls (primary antibody replaced with blocking agent) of pre-culture controls from each foetal sample. Slides for different treatments of the same foetal sample were all processed in parallel in a single experiment.

### Immunofluorescence

Dewaxing, rehydration, antigen retrieval and blocking of endogenous peroxidase and appropriate serum blocking were performed as described above for immunohistochemistry. To detect the primary antibody, slides were incubated with peroxidase-conjugated secondary antibody for 30 min and visualised using Tyramide signal amplification kit (PerkinElmer, Inc.) at 1:50 for 10 min (both steps at room temperature). Antigen retrieval was performed using microwave treatment prior to adding subsequent primary antibodies. Same detection steps using different fluorophores were performed for each primary antibody. Nuclei were counterstained with Hoechst (Thermo Fisher Scientific) diluted in TBS at 1:2000. Tissue sections were mounted with Permafluor (Lab Vision™, Thermo Scientific), and tiled images of the whole tissue section were captured at × 20 magnification using LSM 780 Confocal microscope (Carl Zeiss Microscopy, GmbH). Primary and secondary antibody details used for single and triple immunofluorescent staining are in Additional File 1: Table S1.

### Statistics

Positively stained cells were manually counted and tubular, and tissue areas were measured using Zen 2.3 lite Blue Edition (Carl Zeiss Microscopy, GmbH) software. The interstitial area was obtained by subtracting the tubular area from the tissue area. All tubules per tissue section were included in the analysis. Cell numbers per tubular or tissue area (mm^2^) were calculated and plotted. Percentage in cell number in comparison to control was also calculated. Data were presented as mean +/− SEM. Two-way ANOVA statistical analysis was performed in order to account for inter-individual sample variation using GraphPad Prism 8 software (La Jolla, CA, USA). Statistical significance was set to *p* < 0.05.

### Single-cell sequencing analysis

Single-cell RNA sequencing was previously performed on human prepubertal testis ranging from 1 to 11 years of age [7]. The resulting dataset was interrogated to define the cell states and populations present in the testis of a 7-year-old, whilst MAGEA4 (spermatogonia) and UTF1 (SSCs) expression was extracted from the dataset for individuals aged 1, 7 and 11 years. Data were presented as t-SNE and violin plots.

## Results

### Cisplatin exposure results in differential germ cell loss in human foetal testis

**Fig. 2:**
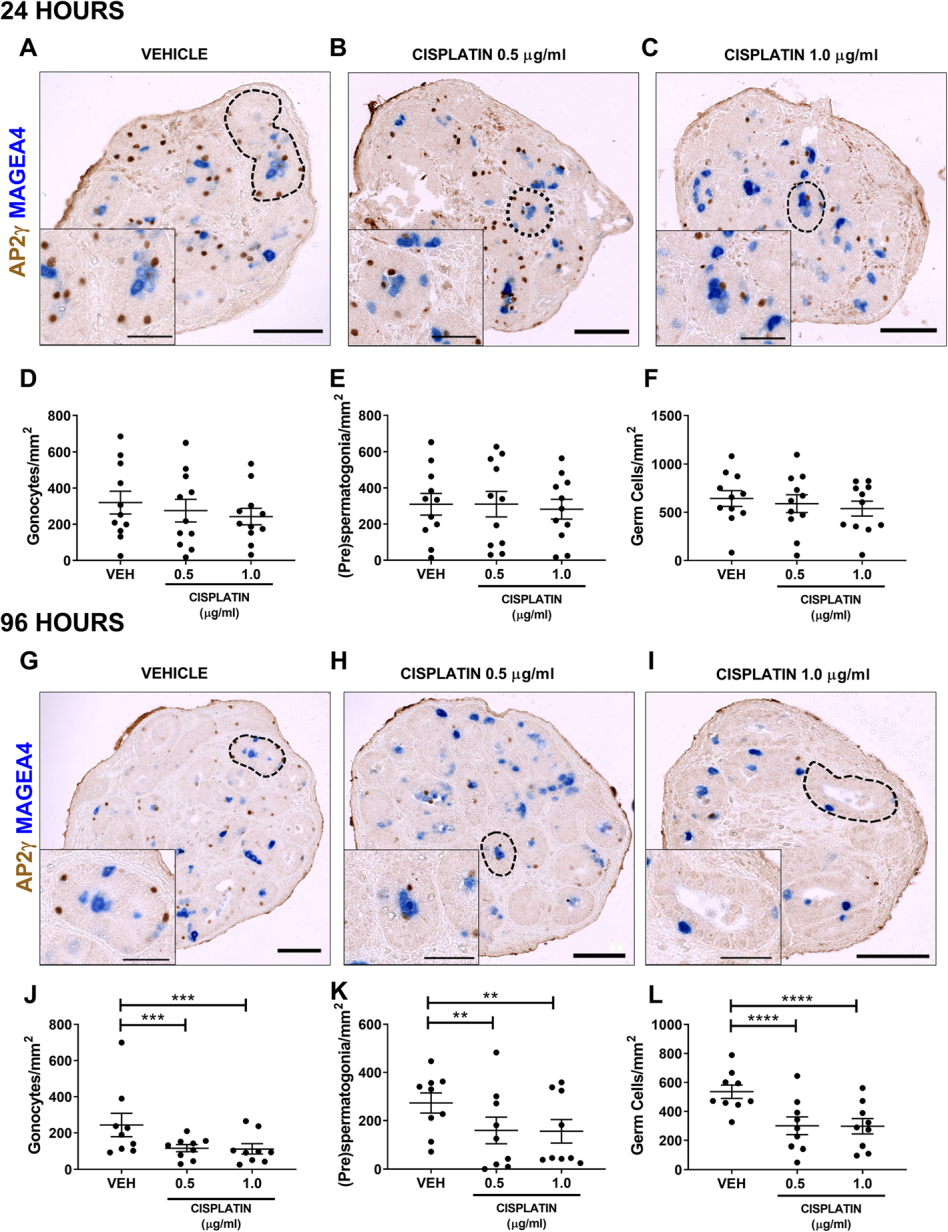
Acute effects of cisplatin exposure on germ cell populations in the human foetal testis. AP2γ^+^ gonocytes (brown) and MAGEA4^+^ (pre)spermatogonia (blue) protein expression in the human foetal testis 24 h (**a**–**c**) and 96 h (**g**–**i**) following exposure to vehicle or cisplatin (0.5 and 1.0 μg/ml). Scale bars represent 100 μm (or 50 μm for insets). Dotted lines outline seminiferous tubules. Quantification of germ cell counts per tubular area (mm^2^) in the human foetal testicular tissues 24 h (**d**–**f**) and 96 h (**j**–**l**) following exposure to vehicle (VEH) or cisplatin (0.5 and 1.0 μg/ml). Germ cell numbers were unaffected 24 h post-exposure, whilst a significant reduction in number of gonocytes, (pre)spermatogonia and total germ cells was observed at 96 h following exposure to 0.5 μg/ml and 1.0 μg/ml cisplatin exposure. Data analysed using two-way ANOVA. **p < 0.01, ***p < 0.001 and ****p < 0.0001. Values shown are means ± SEM and each data point represents an individual foetus (*n* = 9–11)

Human foetal testicular tissues (*n* = 11, 14–22 GW) were exposed in vitro to cisplatin (0.5 and 1.0 μg/ml) or vehicle for 24 h, and germ cells per tubular area (mm^2^) were quantified at 24 and 96 h post-exposure (Fig. 2). Germ cell populations consisted of gonocytes (AP2γ^+^) and (pre)spermatogonia (MAGEA4^+^) (Fig. 2a–c). At 24 h post-exposure, there was no significant difference in either germ cell population in cisplatin-exposed compared to vehicle-exposed testicular tissues (Fig. 2d–f). However, at 96 h post-exposure, germ cell loss was evident for cisplatin exposure at both concentrations (Fig. 2g–i). Quantification showed that both germ cell populations (gonocytes and (pre)spermatogonia) were significantly reduced in cisplatin-exposed testicular tissues (Fig. 2j, k) and overall total germ cell number (gonocytes + (pre)spermatogonia) was reduced by 44% for both 0.5 μg/ml (301 v 536, *p* < 0.0001) and 1.0 μg/ml (298 v 536, *p* < 0.0001) cisplatin exposures (Fig. 2l). Since the degree of germ cell loss was similar with both concentrations of cisplatin, further experiments were focused on exposure to 0.5 μg/ml cisplatin only.

**Fig. 3:**
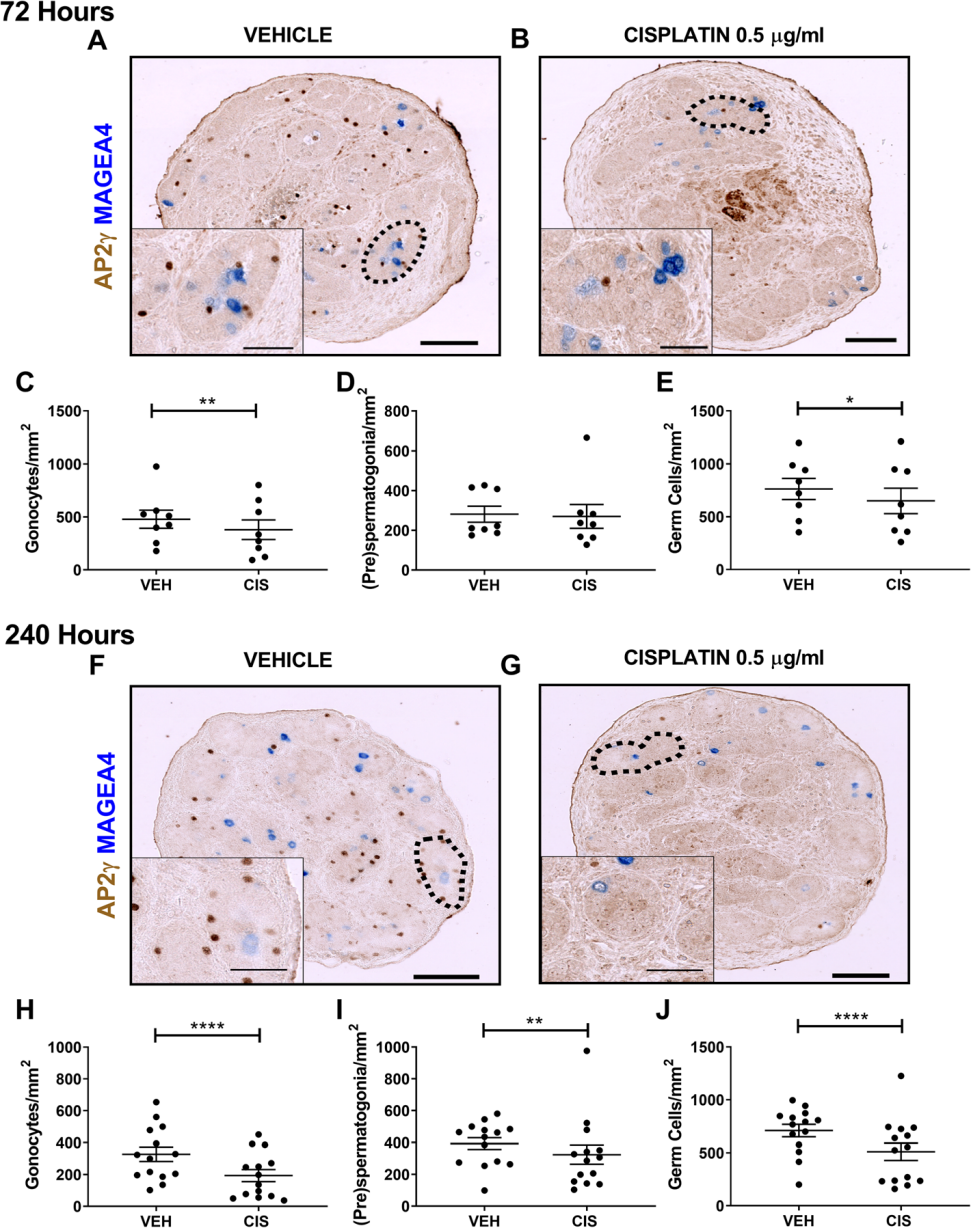
Intermediate and prolonged effects of cisplatin exposure on germ cell populations in the human foetal testis. AP2γ^+^ gonocytes (brown) and MAGEA4^+^ (pre)spermatogonia (blue) protein expression in the human foetal testis 72 h (**a**, **b**) and 240 h (**f**, **g**) following exposure to vehicle or cisplatin (0.5 μg/ml). Scale bars represent 100 μm (or 50 μm for insets). Dotted lines outline seminiferous tubules. Quantification of germ cell subpopulations in the human foetal testis 72 h (**c**–**e**) and 240 h (**h–j**) following exposure to vehicle (VEH) and cisplatin (CIS; 0.5 μg/ml). At 72 h post-exposure to cisplatin, gonocytes and total germ cells were significantly reduced, whilst (pre)spermatogonia were unaffected. At 240 h post-exposure, a decrease in all germ cell populations was observed. Data analysed using two-way ANOVA. *p < 0.05, **p < 0.01 and ****p < 0.0001. Values shown are means ± SEM and each set of data points represents an individual foetus (*n* = 8–14)

In order to determine the timing of germ cell loss for gonocytes and (pre)spermatogonia, we investigated the effect of cisplatin exposure at an intermediate time-point (72 h post-exposure, *n* = 8; Fig. 3a–e). There was a significant reduction in the number of gonocytes in cisplatin-exposed testicular tissues (− 21%, 380 v 479, *p* < 0.01; Fig. 3c). However, (pre)spermatogonial number was unchanged in cisplatin-exposed testicular tissues compared with vehicle control (270 v 281, *p* > 0.05; Fig. 3d). Despite the fact that (pre)spermatogonial number was not affected by cisplatin exposure, the loss of gonocytes was sufficient to cause a significant overall reduction in total germ cell number (− 15%, 650 v 763, *p* < 0.01; Fig. 3e).

Progression of cisplatin-induced germ cell loss was investigated in testicular tissues at 240 h post cisplatin-exposure (*n* = 14; Fig. 3f–j). The reduction in gonocyte (− 41%, 194 v 327, *p* < 0.0001; Fig. 3h), (pre)spermatogonia (− 18%, 324 v 393, *p* < 0.01; Fig. 3i) and total germ cell number (− 37%, 433 v 691, *p* < 0.0001; Fig. 3j) was maintained over the extended culture period.

### Cisplatin exposure reduces cell proliferation in both germ cell populations in human foetal testis

**Fig. 4:**
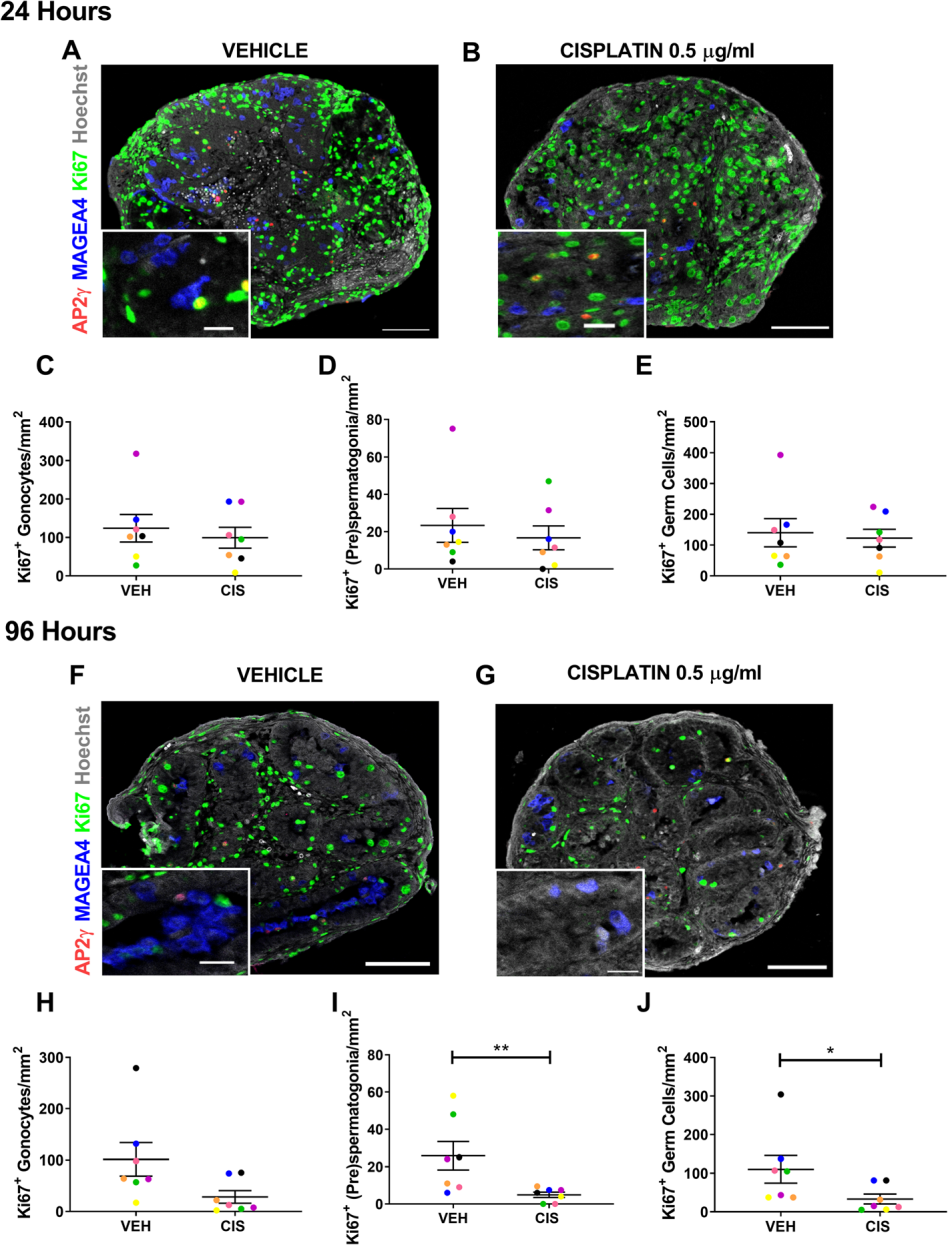
Acute effects of cisplatin exposure on germ cell proliferation in the human foetal testis. AP2γ^+^ gonocytes (red), MAGEA4^+^ (pre)-spermatogonia (blue) and Ki67 (proliferation; green) protein expression in the human foetal testis 24 h (**a**, **b**) and 96 h (**f**, **g**) following exposure to vehicle (VEH) or cisplatin (CIS; 0.5 μg/ml). Scale bars represent 100 μm (or 20 μm for insets). Quantification of germ cell counts per tubular area (mm^2^) in the human foetal testis 24 h (**c**–**e**) and 96 h (**h**–**j**) following exposure to vehicle or cisplatin (0.5 μg/ml). A decrease in proliferating (pre)spermatogonia and total germ cells was observed at 96 h post exposure to cisplatin. *p < 0.05, **p < 0.01. Data analysed using two-way ANOVA. Values shown are means ± SEM and each set of coloured data points represents an individual foetus (*n* = 7)

To investigate further the reason for germ cell loss in cisplatin-exposed testicular tissues, we assessed apoptosis by immunostaining for cleaved caspase 3 (CC3) (Additional File 2: Figure S2). There was no significant difference in total number of apoptotic (CC3^+^) cells in the seminiferous cords of cisplatin-exposed testicular tissues, compared to vehicle controls at either 24 or 96 h post-exposure. Apoptotic cells in the interstitium were also extremely rare in both cisplatin-exposed and vehicle control tissues. Therefore, we assessed proliferation (Ki67^+^) as an explanation for germ cell population loss in cisplatin-exposed testicular tissues at 24 h post-exposure (Fig. 4a, b). At this time-point, there were no significant differences in proliferation of either germ cell population following cisplatin-exposure (Fig. 4c–e). However, at 96 h post-exposure (Fig. 4f, g), there was a reduction in proliferating germ cells in cisplatin-exposed testicular tissues, compared with vehicle controls (Fig. 4h–j). Proliferating (pre)spermatogonia were significantly reduced (− 81%, 5 v 26, *p* < 0.01; Fig. 4i) by cisplatin exposure, and whilst there was a similar trend towards reduction in proliferating gonocytes (− 71%, 28 v 101, *p* = 0.06; Fig. 4e), this did not reach statistical significance. Overall, proliferation was decreased in the total germ cell population (− 70%, *p* < 0.05; Fig. 4j).

These data demonstrate acute and specific loss of germ cells from the seminiferous epithelium of human foetal testicular tissues following cisplatin exposure. Furthermore, there was a differential effect in the timing of germ cell loss, with gonocyte number being reduced at an earlier time-point (72 h) compared to (pre)spermatogonial number (96 h). This germ cell loss was associated with a reduction in the number of proliferating germ cells in the testis.

### Exposure to cisplatin results in long-term germ cell loss in human foetal testis xenografts

**Fig. 5:**
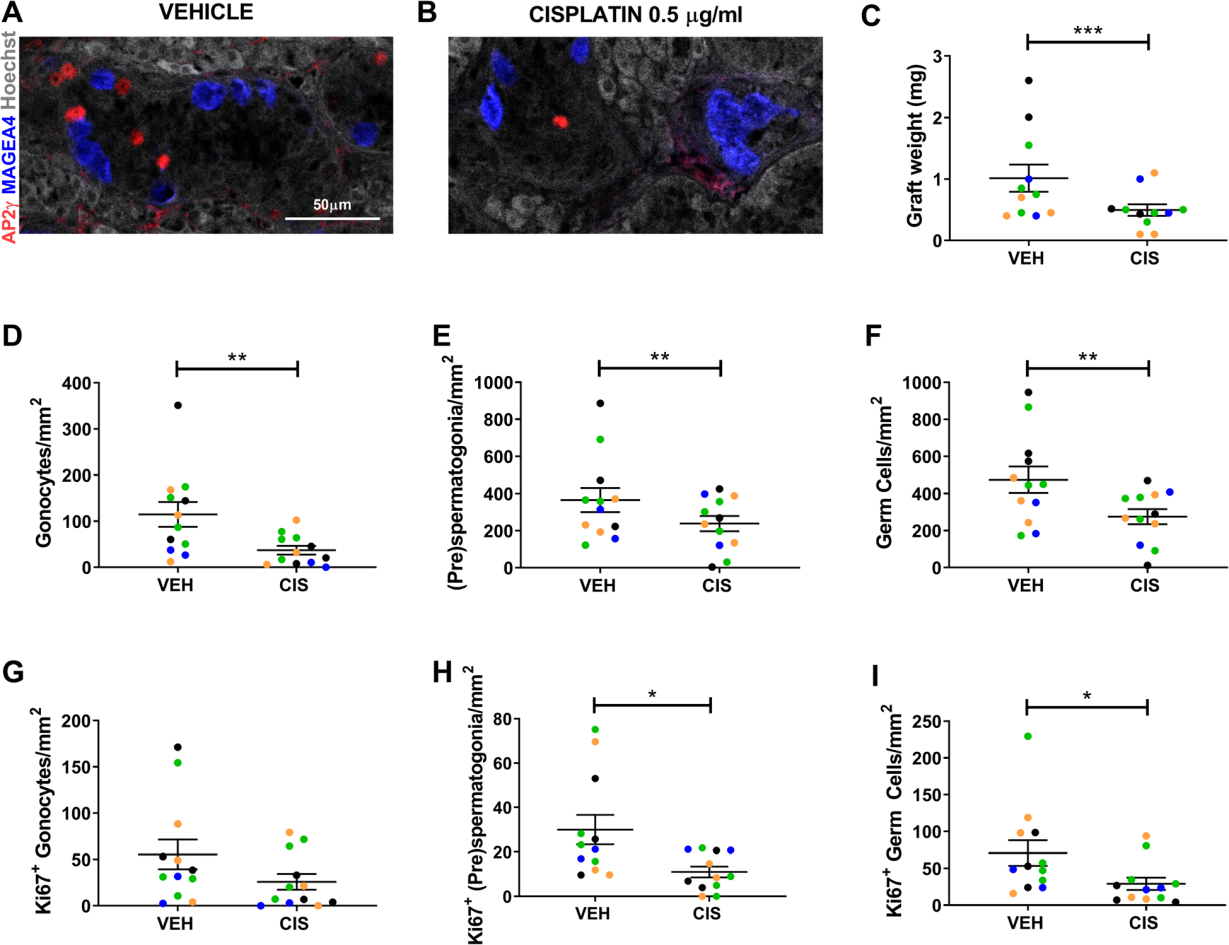
Long-term effects of cisplatin exposure on human foetal testis xenografts. Immunohistochemistry for AP2γ^+^ gonocytes (red) and MAGEA4^+^ (pre)-spermatogonia (blue) in human foetal testis tissues exposed to vehicle (VEH; **a**) or cisplatin (CIS; **b**) for 24 h prior to xenografting (12 weeks). Total graft weight (**c**) was significantly reduced following cisplatin-exposure. Quantification of cell number (**d**–**f**) and proliferation (**g**–**i**) of germ cell populations per tubular area (mm^2^) in xenografts. A significant decrease in all germ cells populations was observed in cisplatin-exposed tissues. Proliferating (pre)spermatogonia and total germ cells were also significantly reduced in cisplatin-exposed tissues after long-term xenografting. Data analysed using two-way ANOVA. *p < 0.05, **p < 0.01 and ***p < 0.001. Values shown are means ± SEM and each set of coloured data points represents an individual foetus (*n* = 4)

To determine whether there is potential for recovery from acute cisplatin-induced loss of germ cells, given the ongoing albeit reduced rate of germ cell proliferation, human foetal (*n* = 4) testicular tissue pieces that had been exposed to cisplatin in vitro were xenografted subcutaneously into immunocompromised host mice for 12 weeks (Fig. 4). Graft retrieval rates were similar for mice xenografted with cisplatin-exposed testicular tissues compared with vehicle controls (90 v 77%, *p* > 0.05). Seminiferous cord structure was maintained and both germ cell populations were identified in vehicle (Fig. 5a) and cisplatin-exposed (Fig. 5b) xenografts. However, mean retrieved xenograft weights (mg) were significantly reduced in the cisplatin-exposed group compared with vehicle controls (− 50%, 0.50 v 1.02, *p* < 0.001; Fig. 5c). Assessment of xenografts at 12 weeks post-grafting revealed a significant reduction in gonocyte (− 68%, 37 v 115, *p* < 0.01; Fig. 5d), (pre)spermatogonial (− 35%, 238 v 366, *p* < 0.01; Fig. 5e) and total germ cell (− 42%, 276 v 475, *p* < 0.01; Fig. 5f) numbers in cisplatin-exposed xenografts compared with vehicle control xenografts. The reduction in graft weight and in total germ cell number in the cisplatin-exposed group could result from either persistent cell loss or a reduction in cell proliferation. Therefore, we quantified proliferation in the germ cell sub-populations (Fig. 5g–i). There was a significant reduction in proliferation of (pre)spermatogonia (− 63%, 11 v 30, *p* < 0.05; Fig. 5h) and total germ cells (− 59%, 27 v 65, *p* < 0.05; Fig. 5i); however, the similar degree of reduction in gonocytes was not statistically significant (− 53%, 26 v 55, *p* > 0.05; Fig. 5g).

Taken together, these data indicate that cisplatin-induced (pre)spermatogonial loss persists in the immature human testis for several months post-exposure and that germ cell proliferation is not sufficiently increased to compensate for this loss.

### Cisplatin-exposure leads to loss of spermatogonial stem cells in the human prepubertal testis

**Fig. 6:**
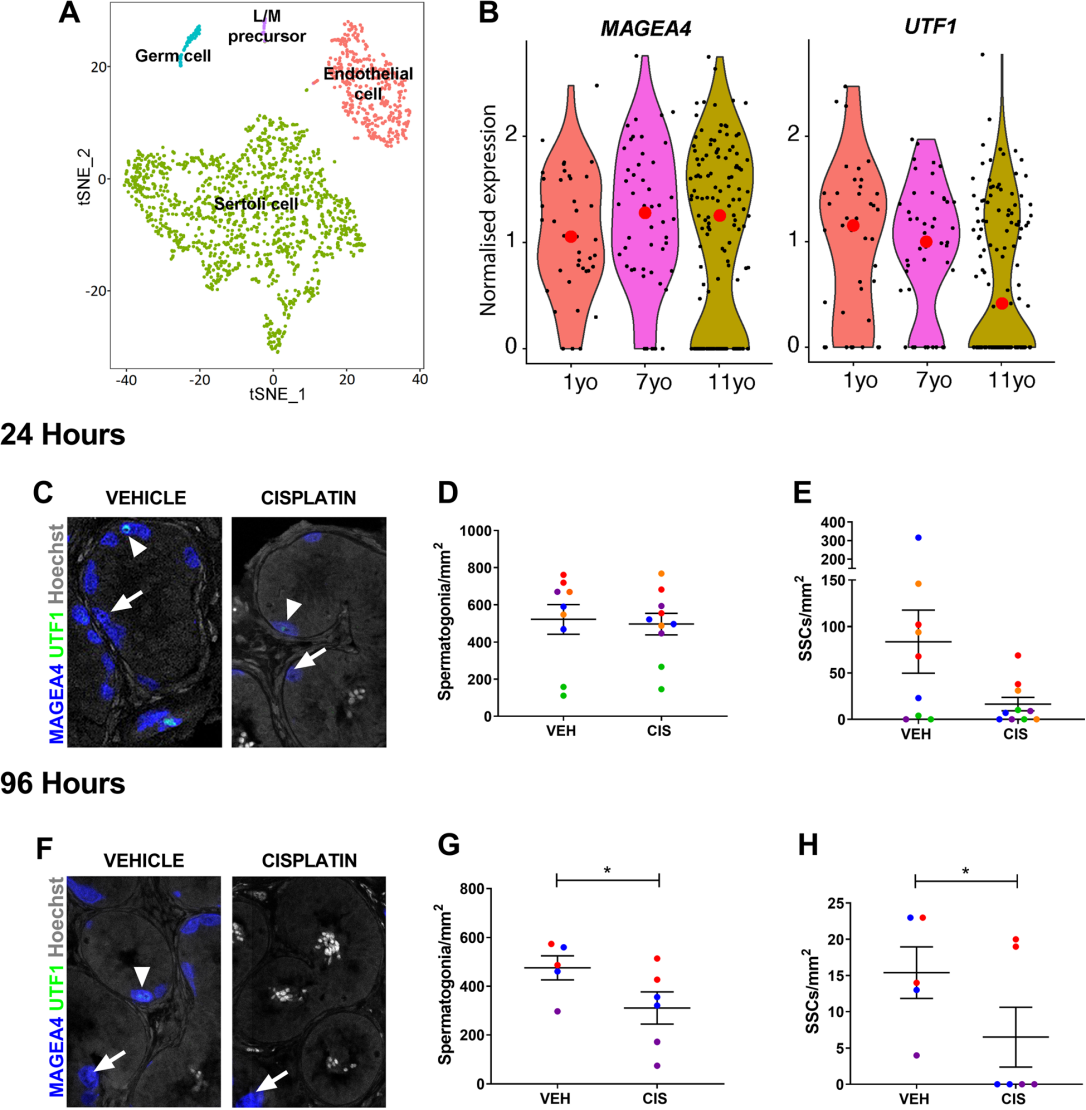
Effects of cisplatin exposure on human prepubertal testis. **a** t-SNE plot of cell populations obtained during single-cell sequencing of testicular tissue of a 7-year-old male. **b** Violin plots for normalised expression of (pre)spermatogonium (MAGEA4) and SSC (UTF1) populations in testicular tissues from 1-, 7- and 11-year-old (yo) males. Red dots represent mean expression. Effect of cisplatin (CIS, 0.5 μg/ml) exposure on MAGEA4^+^ spermatogonia (blue) and UTF1^+^ (green) SSCs in the human prepubertal testis 24 h (**c**–**e**) and 96 h (**f**–**h**) after exposure compared to vehicle (VEH) control. A significant decrease in spermatogonia and SSCs was observed at 96 h post-cisplatin exposure. Data analysed using two-way ANOVA. *p < 0.05. Values shown are means ± SEM and each set of coloured data points (blue and red—1 year, orange—8 years, green—11 years, purple—12 years) represents an individual patient (*n* = 3–5). **c**, **f** Arrows—spermatogonium (MAGEA4^+^), arrowheads—SSC subpopulation (MAGEA4^+^/UTF1^+^)

Spermatogonia in the prepubertal human testis have been shown to express MAGEA4, with a sub-population of spermatogonia co-expressing UTF1 believed to represent spermatogonial stem cells (SSCs) [7]. Given that future fertility is dependent on the survival of the SSC population, we interrogated our recent single-cell transcriptomics data from prepubertal human testicular tissues for MAGEA4 and UTF1 [7]. In tissues obtained from a 7-year-old boy, germ cells were represented by a distinct cluster separate from somatic cell populations (Fig. 6a). MAGEA4 showed relatively stable expression in germ cells of boys aged 1, 7 and 11 years, whilst relative expression of UTF1 was reduced with increasing age (Fig. 6b), probably due to progression towards spermatogonial differentiation [7]. To determine whether cisplatin-exposure in prepuberty results in a similar germ cell (MAGEA4^+^) loss to that described for the human foetal testis and if this included the spermatogonial stem cell (MAGEA4^+^ /UTF1^+^) population, we performed in vitro culture of prepubertal testicular tissue obtained from boys prior to chemotherapy exposure (*n* = 3–5, 1–12 years). Tissues were exposed to cisplatin (0.5 μg/ml) for 24 h, with analysis at 24 (*n* = 5, aged 1, 1, 8, 11 and 12 years) and 96 h (*n* = 3, aged 1, 1 and 12 years) post-exposure (Fig. 6c–h). Cisplatin-exposure did not result in a significant change in the total number of spermatogonia at 24 h post-exposure (536 v 496, *p* > 0.05; Fig. 6d). Overall, SSC number was reduced in prepubertal human testicular exposed to cisplatin at 24 h post-exposure, compared to vehicle-exposed controls, although this was not statistically significant (16 v 75, *p* > 0.05; Fig. 6e). As there was sufficient testicular tissue available from three of the patients (aged 1, 1 and 12 years), we also investigated the effects of cisplatin treatment at 96 h post-exposure. Quantification showed that there was a significant reduction in total spermatogonial number (− 30%, 310 v 446, *p* < 0.05; Fig. 6G) and a reduction in SSC number (− 52%, 6.5 v 13.5, *p* < 0.05; Fig. 6H), compared to vehicle-exposed controls. Importantly, there were no SSCs remaining in the cisplatin-exposed group for 2 out of 3 patients (aged 1 and 12 years) (Fig. 6h).

In summary, these results demonstrate cisplatin-induced germ cell loss in prepubertal human testicular tissues, including a reduction or loss of the SSC population. The degree and timing of spermatogonial cell loss was similar to that seen in human foetal testicular tissues after cisplatin exposure. This provides further validation for the use of the human foetal testis as a relevant model system for studying impacts of exposures during prepuberty, whilst also demonstrating consistent germ cell effects at different key stages of human testicular development.

### Carboplatin induces similar effects to cisplatin on germ cell populations in the human foetal testis

**Fig. 7:**
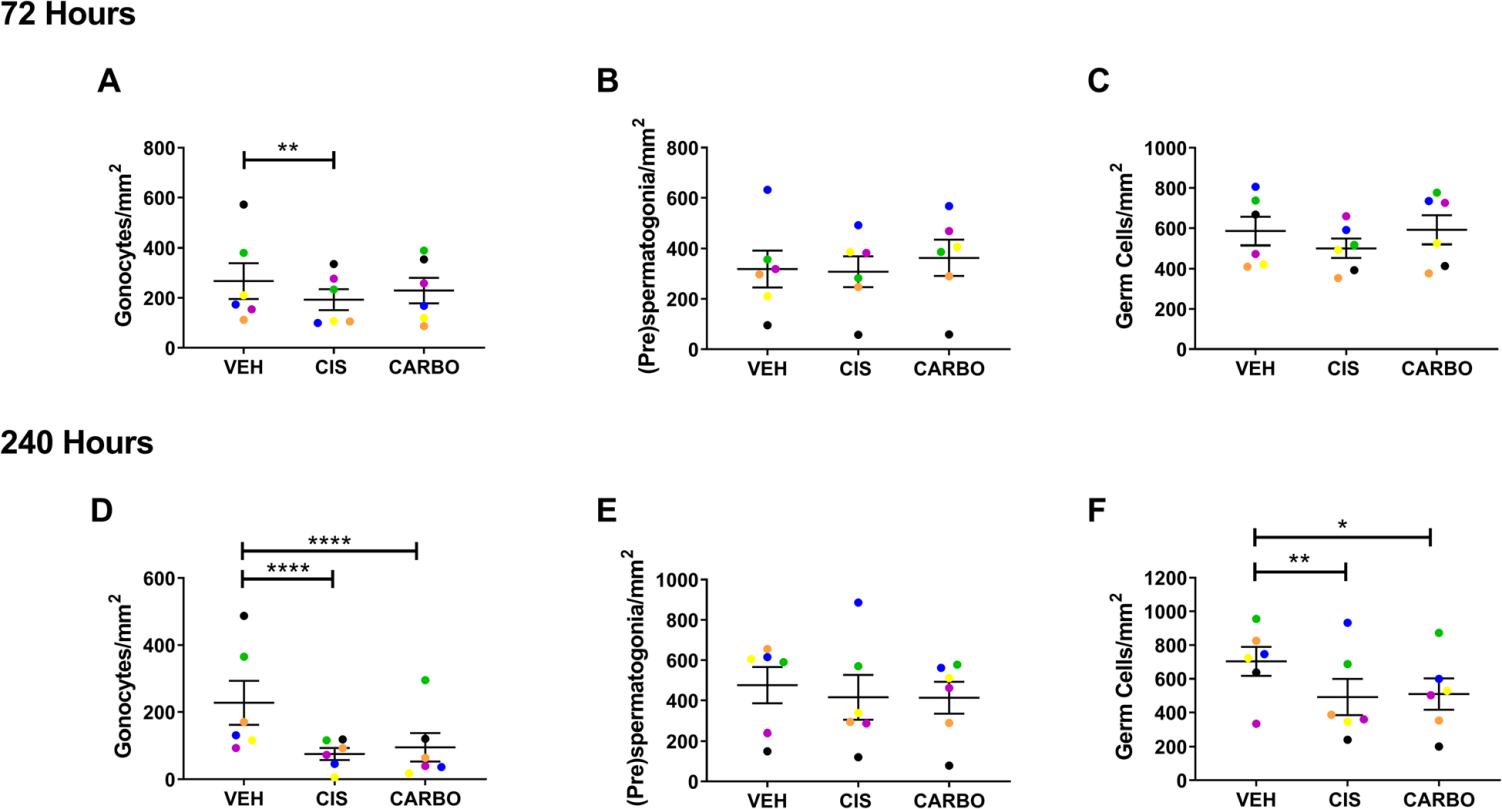
Comparison of cisplatin and carboplatin exposure on germ cell populations of the human foetal testis. Quantification of germ cell subpopulations in the human foetal testis 72 h (**a**–**c**) and 240 h (**d**–**f**) following exposure to vehicle (VEH), cisplatin (CIS; 0.5 μg/ml) or carboplatin (CARBO; 5 μg/ml). At 72 h post-exposure, gonocytes were significantly reduced for cisplatin only. At 240 h post exposure, a decrease in gonocytes and total germ cells was observed following exposure to either cisplatin or carboplatin. Data analysed using two-way ANOVA. *p < 0.05, **p < 0.01 and ****p < 0.0001. Values shown are means ± SEM and each set of data point represents an individual foetus (*n* = 6)

Carboplatin is used in clinical practice as an alternative to cisplatin because of its reported lower risk of side effects. Therefore, we aimed to determine whether carboplatin exposure resulted in a reduction in gonadotoxicity compared with cisplatin, at equivalent human-relevant concentrations. Human foetal testicular tissues (*n* = 6) were exposed in vitro to cisplatin (0.5 μg/ml), carboplatin (5 μg/ml) or vehicle for 24 h: carboplatin was administered at 10 times the concentration to cisplatin in line with relative therapeutic doses used in patients. Germ cells were quantified at 72 and 240 h post-exposure (Fig. 7). At 72 h post-exposure, there was a significant reduction in gonocytes in cisplatin-exposed compared to vehicle-exposed tissues (− 28%, 193 v 267, *p* < 0.01), whilst carboplatin did not result in a significant change in gonocyte number (Fig. 7a). (Pre)spermatogonia and total germ cell number were unaffected after 72 h for both platinum-based agents (Fig. 7b, c). However, at 240 h post-exposure, gonocytes were significantly reduced to a similar extent following exposure to cisplatin (− 67%, 76 v 228, *p* < 0.0001) or carboplatin (− 58%, 96 v 228, *p* < 0.0001), compared with vehicle-exposed controls (Fig. 7d). (Pre)spermatogonial numbers were similar between cisplatin- and carboplatin-exposed testicular tissues after 240 h and were not significantly different to vehicle controls (Fig. 7e). However, total germ cell number was significantly reduced by a similar amount in both cisplatin- (− 30%, 492 v 704, *p* < 0.01) and carboplatin-exposed tissues (− 28%, 509 v 704, *p* < 0.05) compared to vehicle control (Fig. 7f).

Taken together, these results demonstrate that clinically equivalent exposure to carboplatin results in similar effects on the germ cell population of the immature human testicular tissues to those of cisplatin.

## Discussion

The present study, using validated experimental models, demonstrates that exposure to platinum-based chemotherapy agents results in acute loss of germ cells in the immature human testis and that this germ cell loss does not recover several months after exposure. The results also indicate a differential decline in germ cell populations, involving an initial loss of gonocytes followed by a reduction of spermatogonia. Importantly, the effects of carboplatin were similar to those of cisplatin, based on human-relevant drug concentrations.

Cisplatin exposure resulted in a significant reduction in total germ cells in both foetal and prepubertal human testicular tissues in culture. This is in keeping with a previous in vitro study in rodents, which demonstrated a reduction in germ cell number in prepubertal mouse testicular tissues exposed to cisplatin [17]. In that study, there was a > 95% reduction in germ cell number 72 h after cisplatin exposure, whereas the reduction in germ cells in the present study was ~ 70–80% at 96 h post-exposure.

A key finding from the present study was the reduction in the SSC pool in prepubertal human testicular tissues following exposure to platinum-based chemotherapy. SSCs represent the key population required for repopulation and restoration of spermatogenesis in the adult testis following gonadotoxic therapy in primates [18]. Implementation of single-cell transcriptomics has allowed a detailed characterisation of spermatogonial populations present within the adult human testis [19, 20] and several SSC ‘states’ each with a unique transcriptional profile have been described [19]. This approach has also been applied to the human prepubertal testis, indicating the existence of similar SSC signatures, including the expression of UTF1 as a key feature of the SSC pool [7]. The present study demonstrates that exposure to cisplatin in vitro results in a 52% reduction in SSCs (MAGEA4^+^ /UTF1^+^) in the prepubertal human testicular tissues. Importantly, this overall reduction in SSCs includes 2 out of 3 patients (aged 1 and 12 years) in whom no SSCs were identified in the tissue 96 h after exposure. This suggests that fertility may be permanently impaired in some individuals receiving therapeutic doses of cisplatin, consistent with the association of cisplatin exposure with infertility in male childhood cancer survivors [4]. Further studies involving a larger number of patient samples are required to reduce the impact of individual variability and determine whether SSC loss following platinum-based chemotherapy is dependent on age or pubertal status. The present findings are of potential importance for counselling patients on future fertility and for fertility preservation options. Current strategies to preserve fertility in prepubertal boys involve cryopreservation of testicular material [8, 21]. However, the future success of this process to restore fertility later in life is dependent on the preservation of germ cells with stem cell capability of cryopreserved tissues [22]. It is conceivable that some of the surviving (MAGEA4^+^ /UTF1^−^) germ cells in these patients may retain, or acquire, SSC capabilities following gonadotoxic therapy, as has been demonstrated in mouse models of testicular regeneration and repair following exposure to busulphan, a chemotherapeutic belonging to the highly gonadotoxic group of alkylating agents [23].

The impacts of exposure to chemotherapy during childhood on subsequent reproductive function have been investigated in clinical cohorts of childhood cancer survivors [3–5, 24]. These studies demonstrate that exposure to alkylating agents is associated with infertility in males [5]. For cisplatin, the data is less consistent. Exposure to platinum-based chemotherapy was not associated with a reduction in partner pregnancy rates in one cohort of childhood cancer survivors [25], whilst two other studies involving the Childhood Cancer Survivor Study reported a significant reduction in partner pregnancy rates in cancer survivors treated with platinum agents compared to their siblings [4, 5]. Clinical studies involving childhood cancer survivors are limited in their ability to determine the effects of individual agents as they usually involve individuals who have received a variety of treatments, including combined therapies which result in series of small numbers of patients receiving like-for-like treatments. Therefore, studies involving human-relevant experimental systems can be useful to determine the specific effects of an individual agent on testicular germ cells.

The search for effective cancer therapies with reduced side-effects has prompted the development of new generations of drugs with lower cytotoxicity. Therefore, we compared the relative gonadotoxicity of cisplatin with carboplatin and showed that there were minimal differences in the impact on germ cell numbers between these two platinum-based therapies. Exposure to either agent resulted in a significant reduction in germ cell number in human foetal testicular tissues with a similar degree of germ cell loss in cisplatin- (− 34%) compared to carboplatin-exposed (− 26%) testicular tissues at 240 h post-exposure. Although the reduction in (pre)spermatogonial numbers in the cisplatin-exposed group did not reach statistical significance, there was a similar magnitude of reduction to the initial cisplatin-exposure studies and this is likely to be explained by the smaller number of foetal testicular samples used in the cisplatin-carboplatin studies. Importantly, the present work used exposures that reflect differences in the doses given to patients in clinical practice: carboplatin is given at ~ 10 times the dose of cisplatin and therefore the concentrations used in our in vitro treatments reflected this difference. The finding of no difference in relative gonadotoxicity between the two agents is consistent with a recent study comparing in vitro exposure in prepubertal murine testis [26]. This study demonstrated a significant reduction in spermatogonia in both cisplatin- and carboplatin-exposed testis when compared to vehicle-exposed controls with an almost identical proportion of germ cell loss for both drugs [26]. Whilst no advantage of using carboplatin versus cisplatin is demonstrated in relation to gonadotoxicity, the fact that there is no worsening of testicular effects coupled with reported advantages of carboplatin for reducing toxicities in other organs (e.g. ototoxicity) may still support the preferential use of carboplatin. Comparably, replacement of the alkylating agent procarbazine with an alternative (dacarbazine) from the same class is associated with an increased recovery of spermatogenesis without affecting overall patient survival [27]. Overall, these results demonstrate the importance of establishing the relative toxicity on each tissue of potential replacements for current therapies, especially when the replacement is in the same class as the existing agent. Because of the limited availability of human material for research, the analysis had to be performed across broad age groups, ranging from ‘second trimester’ and ‘prepuberty’. Hence, a larger study distinguishing early and late foetal and specific prepubertal periods, e.g. ‘mini-puberty’ in early postnatal life, may provide further insight into gonadotoxic impacts on the testis at specific stages during development.

This study demonstrates the utility of these novel experimental approaches for assessing the acute and long-term effects of chemotherapy exposures on the human prepubertal testicular tissues. In addition, the experimental models may also be used to study the combination of treatments and repeated exposures to mimic regimens used in clinical practice. This would provide direct experimental evidence to complement the findings from clinical studies involving male childhood cancer survivors [5]. Cancer treatments are rapidly evolving to include novel agents including immunotherapy involving monoclonal antibodies and manipulation of specific cell-signalling pathways. For many of these agents, there is no information on long-term reproductive effects. The presently used experimental test systems provide a human-relevant model that can be used to investigate the gonadotoxicity of these emerging cancer therapies.

The results of this study in relation to the sensitivity of the gonocyte population are important and have implications for chemotherapy treatment given to pregnant women. It is recognised that many chemotherapeutics can induce foetal loss due to global toxicity to the mother; however, when foetal loss is not expected, there may still be effects on gonadal function and germ cell survival of the developing foetal testis. During the first trimester of pregnancy, the germ cell population in the foetal gonad consists primarily of gonocytes [16]. During foetal life, there is a gradual transition of the entire germ cell population from gonocyte to (pre)spermatogonia [16, 28]. In the present study, the acute reduction of gonocytes resulting from in vitro exposure to platinum-based chemotherapy agents was associated with a subsequent reduction in (pre)spermatogonia in the human foetal testis. This indicates that the establishment of the spermatogonial population in the foetus may be sensitive to the chemotherapy regimen given to the mother during pregnancy. This is further supported by the finding that the reduction in (pre)spermatogonial number is maintained in xenografted testicular tissues several months after exposure. We have previously shown that human foetal testis xenografts exhibit good preservation of testicular structure, seminiferous cord appearance and histology compared with ungrafted materials and that development from gonocytes to spermatogonia continues during the graft period, similar to age-matched ungrafted control testis [16]. Hence, the xenograft model represents a suitable system to determine the effects of exposure on germ cells in the human foetal testis.

Few studies have investigated the effects of cisplatin exposure in pregnancy. Cisplatin administered to pregnant women for cervical cancer during the second trimester crosses the placenta resulting in concentrations ranging from 23 to 65% (cord blood) and 11–42% (amniotic fluid), compared with those in the maternal circulation [29]. In baboons, administration of carboplatin has been shown to result in foetal serum levels which were ~ 50% of those in the maternal serum and significant malformations were noted in 4% of the exposed foetuses [30]. In rodents, cisplatin accumulated in foetal tissue approximately a week post-administration [31]. Placental transfer occurs through diffusion, although platinum agents also commonly utilise active transport mediated via transporters p-glycoprotein, multi-resistance protein (MRP)-1 and 2 [32], copper influx transporters, ATP7A and ATP7B [33].

Foetal life is a critical period for programming subsequent testicular development and reproductive function [34]. Exposure to a variety of environmental agents and pharmaceuticals during foetal life has previously been shown to result in germ cell loss in rodents and humans [35]. Recent studies have demonstrated that exposure to human-relevant concentrations of acetaminophen (paracetamol) results in a reduction of gonocytes and total germ cell number (including (pre)spermatogonia) in human foetal testicular tissues including acute effects of in vitro exposure and in long-term xenografts [13]. The gonocyte population was predominantly affected in the present study as previously demonstrated for exposure to phthalates [36]. The mechanism for the acute germ cell loss following cisplatin exposure is likely to involve direct damage to the germ cells. We investigated the potential mechanism for cisplatin-induced reduction in germ cell numbers. As previously reported, there is an increase in apoptosis (expressed as percentage of tubules stained with cleaved caspase 3) 8 h prior to the decrease in germ cell number in cisplatin-treated prepubertal mouse testicular tissues [17]. In contrast, the present study suggests that there is no apparent difference in apoptotic cell number at any time point studied. However, activation of cleaved caspase 3 pathway could occur between 24 and 72 h post cisplatin exposure, before the reduction in germ cell number becomes apparent. Interestingly, studies involving acetaminophen- or phthalate-exposure have shown that testicular effects may also involve the associated reduction in testosterone during foetal life [13, 14, 37]. The impact of platinum-based agents on somatic (including Leydig) cell function in foetal and early postnatal testicular tissues requires further investigation.

Early postnatal life is also another crucial period for germ cell development and testicular growth [38]. The hypothalamo-pituitary-gonadal axis is active and the completion of gonocyte differentiation into spermatogonia occurs during this period [38]. As a result, gonocyte loss following platinum exposure during infancy may impact on the establishment of the normal pool of SSCs, and fertility in adulthood. This is of particular importance for malignancies (e.g. neuroblastoma) that can arise shortly after birth or during infancy.

## Conclusion

In conclusion, we show that exposure to human-relevant concentrations of platinum-based chemotherapy results in a reduction in germ cell number, including spermatogonial stem cells, in the immature human testis. We observed a similar germ cell loss for cisplatin and carboplatin, suggesting that carboplatin may not have reduced gonadotoxicity compared to cisplatin. These experimental approaches using human tissues can be broadly applied to determining the relative gonadotoxicity of current and novel cancer therapies, which is important for counselling, modification of treatment regimens and developing strategies to preserve fertility in children with cancer.

## Supplementary Information

**Additional file 1 **: **Table S1.** Antibodies and dilutions used for immunohistochemistry (IHC) and immunofluorescent (IF) staining.

**Additional file 2 **: **Figure S1.** Effect of cisplatin exposure on apoptosis in human fetal testis. Cleaved Caspase 3 (CC3) protein expression in the human fetal testis 24 h following exposure to vehicle (VEH; A) or cisplatin (CIS, 0.5 μg/ml; B). Scale bars represent 100 μm (or 50 μm for insets), arrowheads indicate positively stained cells: (A) cell in the seminiferous tubule, (B) cell in the interstitium. There was no significant change in the number of apoptotic cells within tubules at 24 h (C) and 96 h (D), and no significant change in the number of apoptotic cells in the interstitium at 24 h (E) and 96 h (F) post-exposure to cisplatin. Data analysed using two-way ANOVA. Values shown are means ± SEM and each set of coloured data points represents an individual fetus (*n* = 5).

## Data Availability

The data sets used and/or analysed during the current study are available from the corresponding author on reasonable request. Single-cell sequencing data is available online at: https://humantestisatlas.shinyapps.io/humantestisatlas1/
