# Supplementary figures and images for: Cisplatin and carboplatin result in similar gonadotoxicity in immature human testis with implications for fertility preservation in childhood cancer
Source: BMC Med. 2020 Dec 4;18:374. doi: 10.1186/s12916-020-01844-y (PMC7716476; doi:10.1186/s12916-020-01844-y)

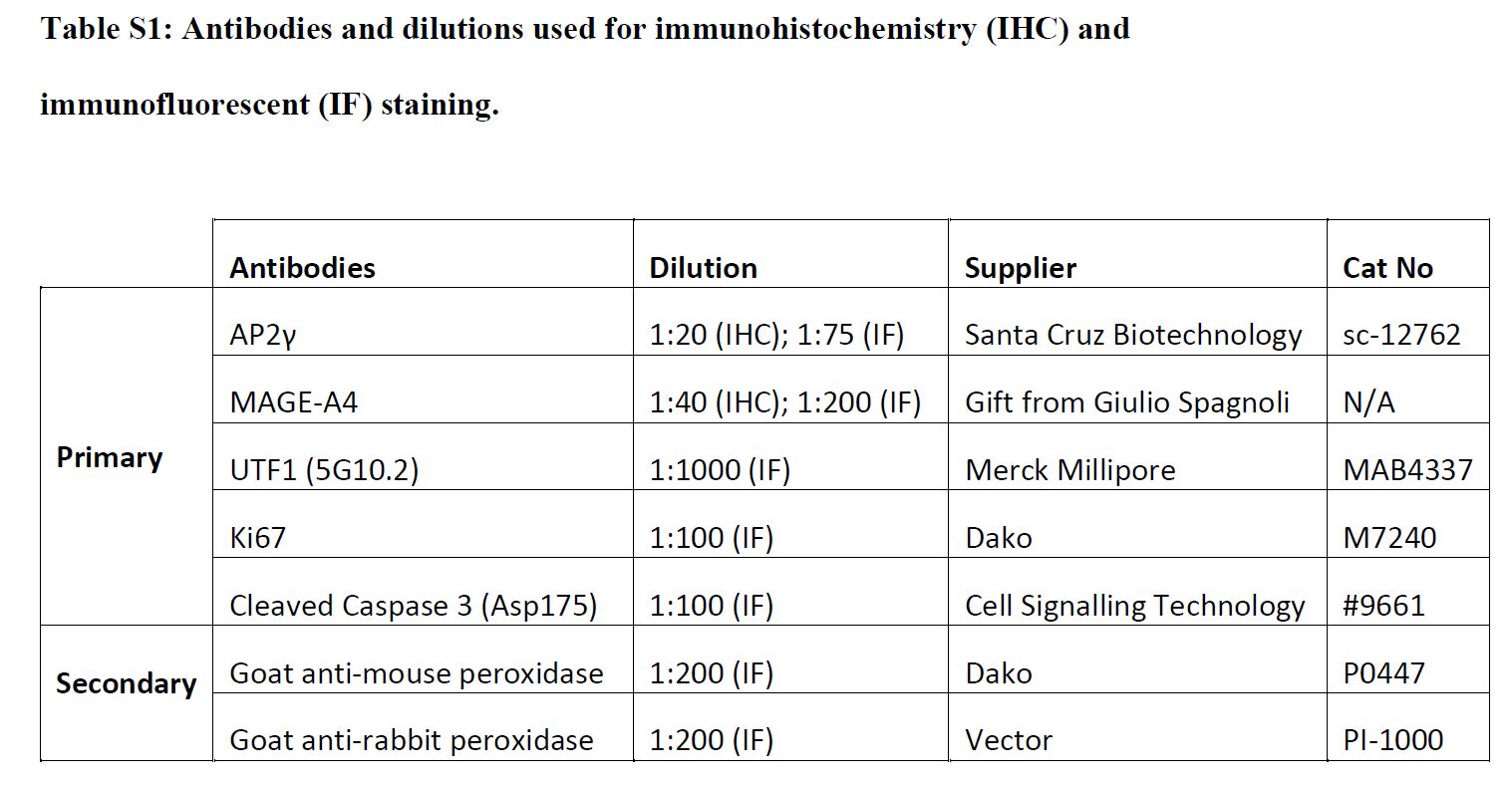

Supplement: Supplementary file 1 — Additional file 1 : Table S1. Antibodies and dilutions used for immunohistochemistry (IHC) and immunofluorescent (IF) staining. [file 12916_2020_1844_MOESM1_ESM.jpg]

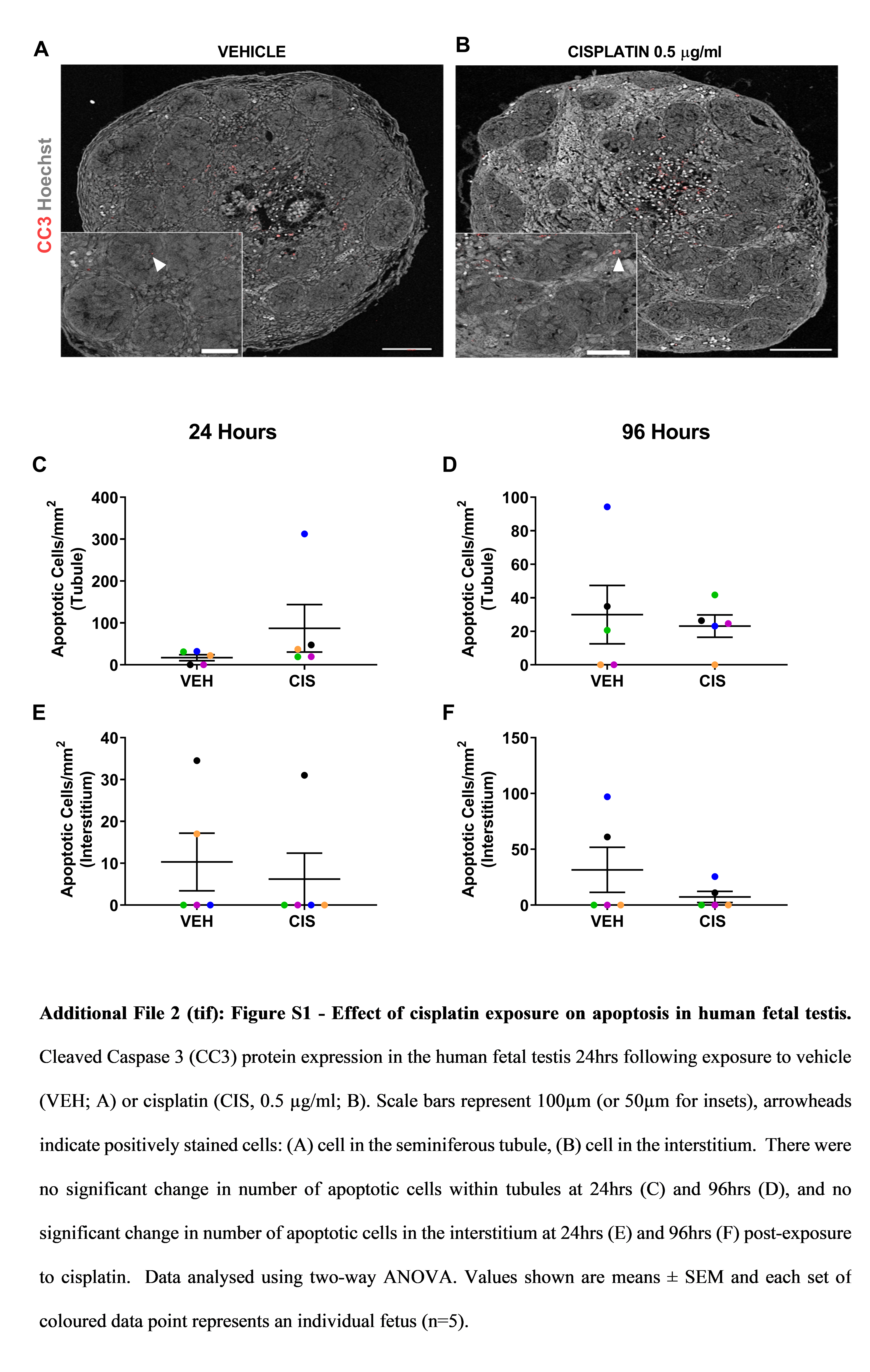

Supplement: Supplementary file 2 — Additional file 2 : Figure S1. Effect of cisplatin exposure on apoptosis in human fetal testis. Cleaved Caspase 3 (CC3) protein expression in the human fetal testis 24 h following exposure to vehicle (VEH; A) or cisplatin (CIS, 0.5 μg/ml; B). Scale bars represent 100 μm (or 50 μm for insets), arrowheads indicate positively stained cells: (A) cell in the seminiferous tubule, (B) cell in the interstitium. There was no significant change in the number of apoptotic cells within tubules at 24 h (C) and 96 h (D), and no significant change in the number of apoptotic cells in the interstitium at 24 h (E) and 96 h (F) post-exposure to cisplatin. Data analysed using two-way ANOVA. Values shown are means ± SEM and each set of coloured data points represents an individual fetus (n = 5). [file 12916_2020_1844_MOESM2_ESM.tif]
